# Dynamic Metabolic State of Tissue Resident CD8 T Cells

**DOI:** 10.3389/fimmu.2019.01683

**Published:** 2019-07-17

**Authors:** Špela Konjar, Marc Veldhoen

**Affiliations:** Instituto de Medicina Molecular, João Lobo Atunes, Faculdade de Medicina da Universidade de Lisboa, Lisbon, Portugal

**Keywords:** T cells, metabolism, tissue resident cells, CD8 T cell, intraepithelial lymphocyte (IEL)

## Abstract

In the past years, there have been significant advances in the understanding of how environmental conditions alone or in conjunction with pathogen invasion affect the metabolism of T cells, thereby influencing their activation, differentiation, and longevity. Detailed insights of the interlinked processes of activation and metabolism can contribute to major advances in immunotherapies. Naive and memory T cells circulate the body. In a quiescent state with low metabolic demands, they predominantly use oxidative phosphorylation for their energy needs. Recognition of cognate antigen combined with costimulatory signals results in a proliferative burst and effector molecule production, requiring rapid release of energy, achieved via dynamically reprogramming metabolic pathways. After activation, most T cells succumb to activation induced cell death, but few differentiate into memory T cells. Of note, some memory T cells permanently occupy tissues without circulating. These, tissue resident T cells are predominantly CD8 T cells, maintained in a metabolic state distinct from naïve and circulating memory CD8 T cells with elements similar to effector CD8 T cells but without undergoing proliferative burst or secreting immune mediators. They continually interact with tissue cells as part of an immune surveillance network, are well-adapted to the tissues they have made their home and where they may encounter different metabolic environments. In this review, we will discuss recent insights in metabolic characteristics of CD8 T cell biology, with emphasis on tissue resident CD8 T cells at the epithelial barriers.

## Introduction

T lymphocytes, especially CD8-expresssing cytotoxic T cells, play a critical role in immune responses to intracellular microorganisms and cancer cells. Naive CD8 T cells are present in the circulation and secondary lymphoid organs (SLOs), where they encounter dendritic cells (DCs) presenting antigens. Naïve T cells may survive for a significant time, only rarely undergoing cell division. One of the underpinning concepts of immunity is the clonal expansion of T cells upon activation. After recognition of cognate antigen via the T cell receptor (TCR) in the context of appropriate co-stimulatory signals, CD8 T cells undergo rapid expansion and traffic from SLOs to the tissues. They differentiate into effector T cells, gaining cytotoxic activity characterized by the ability to release perforin and granzymes. In addition, they can secret large amounts of cytokines, such as TNF and IFN-γ (1). The majority of effector cells generated die by apoptosis, but a small population remains and develops into memory CD8 T cell subsets. Memory T cells were thought to circulate and pass through SLOs, similar to naïve T cells, but in larger antigen-specific numbers and with the intrinsic ability to respond more rapidly to reencountered antigens. These memory T cells are now referred to as central memory T cell (T_CM_) after the recognition that effector memory T cells (T_EM_) circulate through the SLOs as well as the tissues (2). Although initially all memory T cells encountered in tissues were considered T_EM_ cells, more recent work has established a dedicated population of tissue resident memory T cells (T_RM_), which do not circulate through the SLOs and provide a first line of tissue defense at the place of initial antigen encounter (3).

In the past few years it has become evident that CD8 T cell function, differentiation and numeric presence is dependent on nutrient availability, uptake, and processing (4). During different stages of CD8 T cell activation, cells go through dynamic alterations in their metabolic capacity and substrate use. These metabolic changes impact the cells bioenergetic and biosynthetic demands related to substrate uptake, mitochondrial function, and protein and lipid synthesis, ultimately influencing cell division, differentiation and effector capacity (5). In this review, we will discuss the recent findings shedding light on the intertwined relation between metabolic pathways and T cell biology, with focus on CD8 T cells, especially those that have taken residence in peripheral tissues.

## Metabolism of Naive CD8 T Cells

Metabolic demands of antigen inexperienced naive T cell are low. Their quiescent state needs to maintain the ability for base level proliferation only and metabolic activity largely serves to support cell migration and survival upon cells moving through the blood stream, lymph, and SLOs. Antigen inexperienced T cells use oxidative phosphorylation (OXPHOS), which generates an estimated 96% of energy needs (6). OXPHOS is the main source of energy in most eukaryotic cells, most efficiently obtaining energy in the form of adenosine triphosphate (ATP) by oxidizing nutrients using specialized enzymes in the mitochondria (7). OXPHOS can use a variety of substrates such as glucose, amino acids, and fatty acids, converted to acetyl-CoA, which enters the tricarboxylic acid cycle (TCA) cycle.

The quiescent metabolic status of naive CD8 T cells is not a default setting due to the cells lack of receiving any activating signals, but is actively maintained. Naïve T cells receive constant signals from cytokines such as IL-7, critical in sustaining basal levels of nutrient transporters, like GLUT1 for glucose uptake, and expression of anti-apoptotic proteins (Bcl-2), required for the long term survival of naïve T cells (8, 9). In addition to obtaining energy from glucose, naive T cells can oxidize lipids, such as oleate and palmitate (10).

## Glycolysis in Effector CD8 T Cells

The activation of T cells rapidly switches the metabolic programmes from OXPHOS toward aerobic glycolysis, PPP, and glutaminolysis. The shift in metabolism is associated with a change in metabolic transcriptome, with mammalian target of rapamycin (mTOR), hypoxia-inducible factor 1 (HIF1), and c-MYC amongst the most prominent factors with the ability to rewire cell metabolism. In T cells, the mTOR pathway upregulates nutrient uptake (especially amino acids), activates glycolytic pathways and promotes cap-dependent translation. Activated CD8 T cells deficient in mTOR, or CD8 T cells treated with mTOR inhibitor rapamycin, become anergic, cannot proliferate and are incapable of metabolic reprogramming during activation (11). Molecular mechanisms by which mTOR influences T cell metabolism and differentiation are discussed elsewhere (12). In activated CD8 T cells, HIF1 upregulates aerobic glycolysis by promoting the transcription of the enzyme pyruvate dehydrogenase kinase 1 (Pdk1) and lactate dehydrogenase A (Ldha) (13). Another transcription factor required for the increase of glycolysis and glutaminolysis in activated CD8 T cells is the transcription factor c-Myc, transcriptionally regulating GLUT1 expression levels. Deletion of Myc abrogates activation induced proliferation and effector function of CD8 T cells *in vitro* and *in vivo* (14, 15).

Glycolysis is a highly conserved metabolic pathway that, independent of oxygen, converts glucose via a series of enzymatic reactions in the cytosol of cells into pyruvate (16). Despite its name, glycolysis does not solely use glucose, most monosaccharides can be converted into pyruvate. Pyruvate can be transported into the mitochondria and oxidized to generate acetyl-CoA. Alternatively, pyruvate remains in the cytosol and is converted into lactate. Lactate production was thought to occur as a consequence of anaerobic glycolysis, when the coenzyme nicotinamide adenine dinucleotide (NAD) required for glycolysis can be in short supply, but it can be produced as part of aerobic glycolysis (Warburg effect). Lactate is produced upon high-energy demands, such as T cell activation, possibly because of limited availability of NAD. Limited NAD availability may result in a switch to lactate production, which itself supplies additional NAD for continued glycolytic flux. Importantly, the production of lactate does not reduce the amount of pyruvate used for OXPHOS and both aerobic glycolysis and OXPHOS pathways are increased during cell activation (15, 17).

The importance of glycolysis for cytotoxic T cell function was shown using the glycolysis inhibitor 2-deoxyglucose (2DG), resulting in defective T cell cytotoxic capacity and selective reduction of the expression of key effector molecules, including IFN-γ and granzymes (18, 19). Of importance, enzymes involved in glycolysis can make direct contributions to T cell function. Increasing glycolysis capacity upon T cell activation result in the engagement of cytosolic glyceraldehyde 3-phosphate dehydrogenase (GAPDH) in catalyzing the conversion of glyceraldehyde 3-phosphate to D-glycerate 1,3-bisphosphate, releasing it from binding to IFN-γ, thereby enabling its translation by human and mouse CD8 T cells (17, 20).

The reason for lactate production remains uncertain, but the energy needs may be acutely high so that the ATP production from rapid glycolysis alone is more efficient, possibly due to limited amounts of NAD+ required in the respiratory chain (21). Lactate can be oxidized back to pyruvate to be used for OXPHOS in some organs, such as muscle and brain, or can be converted to glucose via gluconeogenesis in the liver to be release back into the circulation. The latter would have the potential to sustain or control high-energy demand processes such as immune responses via the liver and its systemic glucose level maintaining capacity (22). In addition, lactate can have direct immune- and cell-modulating properties (23, 24). Lactate can inhibit the motility of T cells, arresting them at the site of inflammation, thereby focussing the T cell response (25). The latter may contribute to chronic inflammatory disorders, although CD8 T cell cytolytic function is also inhibited by lactate, possibly acting as a safeguard to prevent immunopathology.

Aerobic glycolysis rapidly generates biosynthetic precursor molecules, can function under otherwise adverse hypoxic or acidic microenvironments, entraps T cells at inflammatory sites and may provide systemic control via blood glucose levels (22, 26). Hence, glycolysis may provide several advantages during T cell activation and inflammation and even contribute to immune resolution.

## OXPHOS in Effector CD8 T Cells

**Figure 1:**
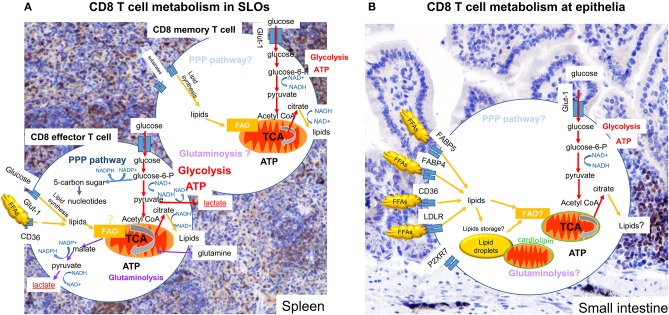
Metabolism of CD8 T cells in SLOs and small intestine. Subtypes of CD8 T cells in different tissues display more or less dependence on particular metabolic pathways and substrates to promote cell survival, activation ability and function. Colored arrows show pathways reported to be used in the cell type indicated, pale text indicates pathways that might be used but have not been clearly defined. **(A)** Effector CD8 T cells in SLOs engage OXPHOS and glycolysis. Pyruvate is mainly converted to lactate, but remains an important metabolite for the TCA cycle. Metabolizing glucose through the PPP can benefit the generation of nucleotides and NADPH, used for lipid synthesis. In addition, glutaminolysis may also be engaged. Memory CD8 T cells use OXPHOS, but also use metabolites provided by glycolysis. **(B)** Tissue resident CD8 T cells (T_RM_) engage OXPHOS and glycolysis. There are increasing indications that OXPHOS is dependent on lipids, either obtained from the local environment or released from internal lipid droplet stores. Furthermore, mitochondria of T_RM_ cells at the epithelia have are more numerous, but have an altered cardiolipon composition, contributing to a poised activation state curtailing OXPHOS potential. The importance of glycolysis for T_RM_ cells remains unknown.

Activation of CD8 T cells does not result in a complete shift from mitochondrial respiration to aerobic glycolysis. OXPHOS levels increase and remain an important ATP contributor to provide the full complement of factors needed for cell proliferation of activated T cells. The increased emphasis on aerobic glycolysis during CD8 T cell activation and parallel increase of OXPHOS may enable other substrates, such as fatty acids and glutamine, to enter the mitochondria to fuel the TCA cycle (14, 15, 27) (Figure 1). T cell activation in the absence of glucose significantly weakens T cell proliferation and function, but this can be partly rescued by supplying pyruvate or galactose. This highlights that mitochondrial respiration remains important in the process of T cell activation. Cells grown in galactose are forced to respire and do not use aerobic glycolysis, generate ATP at a slower rate and produce less IFN-γ compared with cells activated in the presence of glucose (17).

OXPHOS is accompanied by a production of mitochondrial reactive oxygen species (mROS), which increases during T cell activation. mROS plays a role in the activation and subsequent nuclear localization of nuclear factor of activated T cells (NFAT), responsible for the transcription of the T cell growth factor interleukin (IL)-2. In line with this, T cells from Uqcrfs-deficient mice (complex III subunit 5) show decreased activation and diminished clonal expansion upon *in vivo* antigen encounter (28). Furthermore, increased expression of lymphocyte expansion molecule (LEM), which positively regulates the expression of the mitochondrial electron transport chain complex, controlling the activity of OXPHOS proteins and mROS production, resulted in increased CD8 T cell proliferation and function (29). The removal of LEM reduced CD8 T cell proliferation and reduced levels of mROS. Inhibition of mROS in LEM protein overexpressing mice was sufficient to reduce CD8 T cell proliferation and cytotoxicity, confirming the importance of mROS in CD8 T cell activation.

## Metabolism of CD8 Memory T Cells

Although memory formation underpins immune protection and is the basis for vaccination success, how memory T cells are formed is not well-understood. However, their long-term maintenance and ability to respond swiftly upon TCR ligation is, at least in part, due to rewiring of their metabolic pathways. Interestingly, memory formation and improved effector function are often associated with suboptimal T cell activation or metabolite availability, such as reducing mTOR activity and decreased glucose or oxygen levels (12, 30–33). Adenosine monophosphate-activated protein kinase (AMPK) restrains mTOR activity, thereby reducing glycolysis and promoting transition from CD8 effector to memory T cell (34, 35).

There are several different characteristics in metabolic make up between CD8 memory T cells compared to naive and effector CD8 T cells (Table 1). CD8 memory T cells show an enhanced mitochondrial OXPHOS capacity that can provide extra energy required for the proliferative burst. CD8 memory T cells also possess greater mitochondrial mass compared to CD8 effector T cells (36). Although primarily found in non-lymphoid tissues (37), CD8 T_EM_ cells show increased uptake of blood glucose through the glucose transporter-1 (Glut-1) and rely on glycolysis as well as OXPHOS to meet their energy demands (36, 38). As such, although basic energy requirements are reduced, CD8 T_EM_ cells seem metabolically wired in similar fashion as effector T cells. CD8 T_CM_ cells on the other hand, predominantly depend on OXPHOS for both their differentiation and maintenance. They take up lipids and glucose from blood, for lipid synthesis, and use FAO as originally described for all memory CD8 T cells (39, 40).

**Table 1:** CD8 T cells subset with their identifiable markers, effector, and metabolic status.

**Subset of CD8 T cells**	**Naive**	**Circulating memory (T_**cm/EM**_)**	**Effector**	**Tissue resident**
Identity markers	CD44^1^° CD62L^+^ CCR7^+^ CD103^−^ CD69^−^	CD44^hi^ CD62L*^+/−^* CCR7^+/−^ CD103^−^ CD69^−^	CD44^hi^ CD62L^−^ CCR7^−^ CD103^−^ CD69^+^ (early)	CD44^hi^ CD62L^−^ CCR7^−^ (CD49a^+^), CD103^+^, CD69^+^
**Effector status**				
•Immune function	Surveillance SLO	Surveillance of SLO/SLO + tissues	Search and kill infected or tumorigenic cells of all tissues	Surveillance of tissues previously infected
•Cell status	Quiescent	Quiescent	Active	Poised
•Secretory vesicles (Grzm)	Absent	Low	High	High
•Cytotoxicity (TCR/CD28)	TCR + CD28	TCR + CD28	TCR	TCR or cytokines
**Metabolic status**				
•Status	Quiesent	Primed	Active	Arrested
•Nutrient uptake	Basal	Basal	High	High(?)
•SRC	High	High	Low	Low(?)
•Glycolyisis	Basal	Basal	High	Basal(?)
•OXPHOS	Basal	Basal	High	Basal
•Lipid metabolism	+	++	++	++
•Glutaminolysis	–	–	+	?
•Biosynthesis	Minimal, no net growth	Synthesis/oxidation balance, no cell proliferation	High, Cell growth, proliferation	No net growth/cell proliferation

*The table highlights the main characteristics, in common and setting apart, the CD8 T cell subsets; naïve, effector, circulating memory, and tissue resident memory*.

## Lipid Metabolism in Circulating CD8 Memory T Cells

CD8 memory T cells depend less on glycolysis and mainly rely on oxidative phosphorylation and lipid metabolism (36). A role for fatty acid metabolism in CD8 memory T cells was first suggested in mice that lack tumor necrosis factor (TNF) receptor-associated factor 6 (TRAF6), which show similar CD8 T cell activation and expansion but defective CD8 memory T cell generation (34). Although TRAF6 is implicated in several pathways, in CD8 T cells it stimulates AMPK while inhibiting mTOR signaling, thereby increasing fatty acid oxidation (FAO) (41). Upon activation T cells acquire extracellular glucose, which is used to synthesize lipids, can be stored in lipid droplets and subsequently used in FAO (39, 40, 42–44) (Figure 1). Furthermore, memory CD8 T cell development depends on cell intrinsic activity of lysosomal acid lipase A (LIPA) to mobilize fatty acids for FAO (39). The role of lipids stored in droplets during the effector phase remains unknown. Processes of lipolysis and autophagy can degrade lipids for substrate use in metabolic processes (45). Under optimal conditions, such as in SLOs, lipolysis may not be critical. The appearance of lipid droplets *in vitro* takes place within the first 24 h after activation (44). However, upon nutrient deprivation, such as encountered in tissues during inflammation or in the tumor environment, autophagy and lipolysis could become an important contributor to energy demands.

Although FAO is important for memory formation, established memory T cells contain few lipid droplets compared with effector T cells (39, 44). Memory T cells are not known to increase the uptake of lipids from their surroundings, but can use alternative sources such as glycerol, to generate lipids (43, 46) (Figure 1). This suggests that the increased potential of memory T cells for OXPHOS is not explained by FAO, confirm recent results using carnitine palmitoyltransferase I (CPT1)-deficient cells, which cannot generate acetyl-CoA from long chain fatty acids (47).

## Tissue Resident CD8 T Cells

In addition to circulating memory T cells, a more recent subtype of memory T cells, called resident memory T cells (T_RM_) has been described. T_RM_ cells are memory T cells that do not circulate and are predominantly found in non-lymphoid tissues (48, 49), although they have been reported in SLOs (50). In mice, the epidermis, forming the top layer of the skin, is home to specialized T cells, expressing TCRγδ. This population develops during embryogenesis and homes to the epidermis. They have a type 1 immune profile with the ability to produce IFN-γ. The small intestine are another tissue forming a large interfaces between the environment and the body and endowed with a specialized population of CD8 T cells that occupying the very top layer of the tissue, the intraepithelial lymphocyte (IEL) compartment. Similar to the epidermal compartment, innate-like CD8 T cells occupy the murine small intestine early in life, predominantly specialized TCRγδ CD8αα homodimer-expressing T cells with a type 1 immune profile, which home specifically to the IEL compartment. In humans, the IEL compartment is mainly composed of TCRαβ T cells with γδ T cells reported in the minority (51). Induced IELs or CD8 T_RM_ cells intercalate with innate-like small intestinal and skin IELs, predominantly expressing TCRαβ and the CD8αβ heterodimer (52, 53). The population of CD8 T_RM_ possess a distinct genetic signature compared with circulating CD8 T cells and are often defined by cell surface expression of CD69, CD103, and CD49a (54). The expression of CD49a and CD103 is indicative of cell interactions with collagen and E-cadherin epithelial tissues (54). Expression of CD69 together with expression of NK cell inhibitory receptors (CD244), and high levels of granzyme B marks CD8 T_RM_ cells as semi-activated T cells (53).

T_RM_ cells are metabolically highly active, continuously scanning the tissues for invasion using migration and long dendrite-like protrusions (52, 55), in line with their semi-activation status. Yet, their development is more in line with CD8 T_CM_ cells than T_EM_ cells (35). T_RM_ cells protect the host through rapid responses upon re-exposure to previously encounter pathogen as well as contribute to immune responses against newly encountered microorganisms via bystander activation (3, 56). Activation of CD8 T_RM_ cells influences the surrounding tissue cells, such as a number of broad acting antiviral and antibacterial genes (57), increasing local defenses and decreasing susceptibility to invasion. Release of inflammatory molecules such as IFN-γ, results in the recruitment of additional myeloid and lymphoid cells, maturation and migration of local dendritic cells and activation of natural killer cells (58). In addition to sentinels of microbial invasion, T_RM_ cells bridge the adaptive immune system with the innate immune system (57, 58).

The integration of CD8 T_RM_ cells within tissues involves adaptation to the local environment compatible with their role in clearing potential pathogens, which necessitate changes in T cell metabolism for their maintenance as well as functional potential. A prime feature of T_RM_ cells is their ability to deeply penetrate tissues and their long-term maintenance within it. T_RM_ have to adapt to the new environment of periphery, which can differ in oxygen levels, nutrient availability, acidity, competition for resources in comparison to the environment of lymphatic organs (59, 60).

CD8 T_RM_ cells, not circulating CD8 T cells, express high levels of the purinergic receptor P2RX7, triggered by extracellular nucleotides, associated with tissue damage and exported by activated T cells (35, 61, 62). P2RX7 is involved in the establishment, maintenance and functional properties of CD8 T_CM_ and T_RM_ cells (35). Although tissue damage and stress have been associated with the activation of tissue integrated CD8 T cells, engagement of P2RX7 can result in the selective cell death of CD8 T_RM_ cells. The balance between activation and cell death is carefully orchestrated and context dependent, low concentrations are able to activate T cell and high concentrations result in cell dead (63). However, P2RX7 can be activated directly by ATP, or indirectly via NAD-dependent ADP-ribosylation by the ecto-ADP-ribosyltransferase ARTC2.2. The concentration of NAD thereby lowering the threshold of ATP concentrations required inducing apoptosis. This may be part of protecting tissues from aberrant immunity in the absence of cognate antigen reencounter since TCR stimulation of CD8 T_RM_ cells reduces P2RX7 expression and susceptibility to cell death. Recent data highlight the importance of both receptors in studying tissue resident T cells, the isolation process resulting in tissue damage and release of ATP and NAD, can causing reduced T_RM_ cell viability and compromises functional assessment (62, 64).

Aberrant regulation of IELs can compromise barrier function and increase susceptibility to infection and immunopathology (44, 65), which can ultimately contribute to inflammatory bowel disease and psoriasis (66, 67). These findings underscore the physiological significance of tissue integrated CD8 T cells in tissue homeostasis and disease. Due to different environmental conditions and their semi-activation status, the metabolic wiring of T_RM_ cells has to be adjusted, the details of which may depend on the host tissue. T_RM_ cells posse some similarities with T_CM_ and T_EM_ cells. We will here discuss some of the recent finding concerning T_RM_ cells and their metabolism.

## CD8 T_RM_ Cells and Lipid Metabolism

Lipids are, in comparison to SLOs, abundant at epithelial barriers where T_RM_ cells persist (59, 60). IELs, in skin and intestine, have adapted to a lipid-rich microenvironment. Early on, it was recognized that intestinal T_RM_ cells express required surface molecules to obtain lipids from the extracellular space, including low density lipid receptor (LDLR), ApoE, scavenger receptor CD36, and fatty acid biding proteins (FABP) 4 and 5 (Figure 1), suggesting an important role for lipid metabolism (68). FABP proteins are involved in FFAs fatty-acid influx and transfer from cytosol to mitochondria for the purpose of β-oxidation (69). In addition to regulating fatty acid influx, in macrophages FABP4 is involved in the nuclear factor-κB (NF-κB) pathway and stimulates pro-inflammatory effector function such as production of cytokines and inducible nitric oxide synthase (iNOS). Furthermore, FABP4 reduces cholesterol ester accumulation via inhibition of peroxisome proliferator-activated receptor-γ (PPAR-γ) pathways and is involved in integrating lipid signals to organelle responses, especially the endoplasmic reticulum (ER) (70). FABP5 is highly expressed in epidermal cells, but is found in many organs. Its function is to enhance lipolysis (71). Due to compensation mechanisms, combined deletion for FABP4/5 show a much stronger phenotype. Adipocytes and macrophages from double deficient mice have an altered lipid profile, in favor of shorter-chain fatty acids (C14). These changes result in higher glucose uptake, AMPK activity, and fatty-acid oxidation (72).

How skin CD8 T_RM_ persist and function in a lipid rich environment remained elusive (59, 60). More recently, Pan et al. (43), using a mouse model of cutaneous immunization with Vaccina virus, showed that CD8 T_RM_ cells in the skin adapt to utilize lipid metabolism using free fatty acids (FFA) obtained from the surrounding microenvironment for their endurance as well as effector function. Activation of CD8 T_RM_ cells fosters a transcriptional program that features notable increased expression of molecules facilitating exogenous FFA uptake and storage. Compared with naïve and circulating memory CD8 T cell subsets, sCD8 T_RM_ cells, in mouse and human, were able to express high levels of FABP4/5 and CD36 and lipoprotein lipase (LPL) (43). T cell specific deletion of FABP4/5 showed an impairment in FFA uptake in CD8 T_RM_ cells, limiting OXPHOS potential and reducing the survival of skin CD8 T_RM_ cells but not circulating memory CD8 T cells (43).

Intestinal CD8 T_RM_ cells show a similar transcriptional programme to skin T_RM_ cells, with pathways involved in FFA and cholesterol ester synthesis increased compared with naive and memory CD8 T cells (44, 68). Furthermore, intestinal IELs store accumulated FFA in lipid droplets (44), from which FFA can be made available for FAO via autophagy or via mitochondria tethered to the lipid droplet (73). These observations suggested a reliance of IELs on FAO. However, the accumulation of lipids is a characteristic of activated T cells and does not appear unique to IEL (36, 39, 44). The conditions under which FFA are made available and used for FAO remain unclear with IELs performing basal OXPHOS without additional capacity upon mitochondrial uncoupling (43, 44, 55). Yet, short-term culture of skin IELs with FFA or intestinal infection with *Salmonella* does result in a modest increase in OXPHOS (43, 55). The data suggests that the trigger that makes available FFA from lipid droplets is likely the same that releases additional OXPHOS potential in T_RM_ cells.

Transcriptomic analysis of IEL during intestinal *Salmonella Typhirum* challenge compared to steady state IELs revealed, once more, increased expression of genes involved in metabolism (55). *Salmonella* infection in the small intestine resulted in increased aerobic glycolysis, glucose uptake, as well as OXPHOS by IELs, similar to effector CD8 T cells (17, 36). In addition, IELs altered their immunosurveilance behavior upon infection, suggesting that the IEL semi-activation status can be further enhanced upon microbial encounter. In support of this, the use of the glycolysis inhibitor 2DG as well as mTOR inhibitor rapamycin resulted in increased *salmonella* burden and invasion. Although 2DG treatment would target many cells involved in the respond to enteric infection (74), GLUT1-deficient innate-IELs revealed the requirement for glycolysis in IELs. However, upon *Salmonella* infection a proliferative response was not detected (55). Collectively, IELs store large amounts of energy in the form of lipids, the signals resulting in the release of these remain unknown, but in line with effector T cells, IELs require both glycolysis and OXPHOS for their effector functions.

## CD8 T_RM_ Cells and Mitochondria

Lipid droplet associated mitochondria are biochemically distinct from non-associated cytoplasmic mitochondria, with the later primarily using pyruvate as a substrate (75). These recent data could explain the observations that FAO and fatty acid synthesis seem to take place at the same time within memory T cells (39), but individual mitochondria can only perform one or the other. Interestingly, mitochondria in brown fat associated with lipid droplets, were shown to have reduced FAO capacity compared with cytoplasmic mitochondria. This suggest they are involved in lipid storage under steady state conditions and, upon exposure to the environmental cue of cold, initiate FAO (75).

Mitochondria actively contribute to T cell activation and circulating T cell memory formation, their fission and fusion determining energy production (76). Furthermore, P2RX7 is involved in metabolic function via stimulation of AMPK in CD8 effector T cells, increasing glucose and fatty acid uptake and OXPHOS, and by promoting mitochondrial fusion and reorganization, affecting the development of CD8 T_CM_ and CD8 T_RM_ cells (35). Unexpectedly, detection of mitochondria using mitotracker dyes, often equated to represent mitochondrial mass, or nonyl acridine orange, which binds to cardiolipins, in T cells, suggested very low levels of mitochondria to be present in IELs (innate-like as well as T_RM_ cells) compared with circulating CD8 T cells, with reduced mitochondrial membrane potential and ROS production (44). These observations were at odds with the active scanning behavior of barrier IELs, as well as their high expression level of P2RX7, the absence of which results in reduced OXPHOS potential but similar aerobic glycolysis (35, 77). Electron microscopy analysis revealed increased numbers of mitochondria to be present in IELs compared with circulating CD8 T cells, albeit of a smaller average size (44). Although the exact binding properties of dyes remains elusive, it suggested marked changes in mitochondria of CD8 T_RM_ cells at epithelial barriers.

IEL mitochondria were found to have an altered cardiolipin make up, enriched in longer and more unsaturated species. Additional experiments using T cells deficient in Tafazzin, an enzyme involved in cardiolipin metabolism, indicated that changes in cardiolipins in line with circulating T cells are required for IEL activation (44, 78). Failure to alter the cardiolipin makeup restricts swift IEL proliferation and effector function, reducing microbial containment capability resulting in an increased microbial burden. In addition, the data also suggested that other changes in IELs may contribute to the absence of mitochondrial detection and possibly energy capacity, since changes in cardiolipin composition did not explain the absence of Mitotracker dye staining under steady state conditions. Whether mitochondria in IELs directly associate with lipid droplets and if their detection is masked by their subcellular location remains to be determined. These findings uncovered an alternative mechanism of mitochondria to control cellular activity, which appear particular to epithelial-resident CD8 T cells.

## CD8 T_RM_ and AhR Metabolism

Lipid metabolism may involve a factor shared between epithelial T cell subsets, critical for the maintenance of IELs but not expressed in circulating T cells, the arylhydrocarbon receptor (AhR) (52, 65, 79, 80). AhR has been linked with cholesterol biosynthesis in hepatocytes as well as attenuating the expression of key fatty acid synthesis genes (81). The transcriptional activity of AhR is the result of ligand engagement in the cytosol (82). The absence of AhR results in alterations in intestinal microbial composition and acute sensitivity to intestinal injury, in line with the role of IEL in controlling the microbiota and regulating epithelial cell turnover and wound repair (65). The identity of the ligand remains unknown, but can include substances derived from food, light, and microorganism (65, 83, 84). However, AhR ligands are lipophilic and likely enriched in lipid-rich tissues. The transcriptional activity of AhR involves the production of metabolic enzymes, cytochrome P450 of the first family (Cyp1), including in T cells (85), involved in the metabolism of polyunsaturated fatty acids and arachidonic acid.

Although, the functional roles of FABP4/5 remain to be defined, their activity may be juxtaposed to that of AhR, the activity of which can dampen psoriasis-like symptoms (66). Since lymphocytes expressing AhR are enriched in tissues, besides CD8 T_RM_ cells, ILC3 and T_H_17 cells, it is tempting to speculate that the AhR system provides a specific advantage in the tissue environment, not required in SLOs. Whether AhR is involved in the assistance of specific metabolic pathways generating energy or protection from metabolic factors encountered in tissues, or generated because of specific metabolic pathways, remains to be determined.

## CD8 T_RM_ and Systemic Metabolism

In addition to IELs primary role to provide a first line of defense against invading microorganisms and tissue homeostasis, recent data suggests a potential role in systemic metabolism. In mice deficient in integrin-β7, which can pair with integrin-α4 (forming CD49d) or αE (forming CD103), immune cell homing to tissues is reduced. Intergin-β7-deficient mice lack natural IEL and are metabolically hyperactive (86). Consequently, these animals are resistant to obesity, hypertension, diabetes, and atherosclerosis when fed a high fat and high sugar diet. IELs express the glucagon-like peptide-1 receptor (GLP-1R) (87). IEL function, release of cytokines and antimicrobial factors, depends on the expression of GLP-1R, its absence resulting in dysregulated intestinal gene expression, an altered microbiota composition, and enhanced sensitivity to colitis, similar to AhR-deficiency with a link to psoriasis (88). GLP-1R, binding GLP-1, is known to be expressed on pancreatic β-cells and brain, its stimulation controlling blood glucose levels and appetite. Its stimulation converting ATP to cyclic adenosine monophosphate (cAMP), reducing the activation and function of IELs, but not that of circulating CD8 T cells (87).

Recent data indicates that the GLP-1R pathway in IELs can determine systemic metabolic capacity, whereby GLP-1 is released from enteroendocrine L-cells in gut epithelium (86). GLP-1 release is increased by sugars and bile acids in the intestinal lumen and in response to neuronal stimulation and inflammation (89). These data suggest that IELs also function as a metabolic and gut-health rheostat, their activity determined by the nutritional and inflammatory state of the organism, maintaining tolerance at the intestinal barrier when symbiotic bacteria produce carbohydrates and bile acids. If GLP-1 levels are limited to reach the blood stream, IEL capture will reduce the availability to stimulate β-cells and the release of insulin, increasing blood glucose levels and activity, as well as to the brain, increasing appetite. The physiological role of this process requires further scrutiny. Since inflammatory signals stimulate L-cell GLP-1 release (89), this could inhibit IEL activity and potentially aggravate intestinal inflammation. Although of potential benefit in times of scarcity, current food composition and ready availability may be detrimental to health.

## Conclusions

In recent years, T cells metabolic characteristic in relation to their activation stage, differentiation and function have been more closely studied. In CD8 T cells, there are clear differences between the metabolic pathways used between naïve, memory and effector cells (Table 1). In addition, between the three identified memory T cell subsets, T_EM_, T_CM_, and T_RM_ cells, there are communalities and differences affecting cell development, maintenance and function. How the development of these memory subsets are fine-tuned, with initial differences between T_RM_ cell development compared to T_EM_ and T_CM_ cells reported, remains incompletely understood. CD8 T_RM_ cells have characteristics of effector T cells, with increased expression of transcripts for proteins involved in metabolism and effector proteins such as granzymes, active cellular migration, as well as uptake of FFA and storage in lipid droplets, but without active proliferation or secretion of effector molecules such as IFN-γ. The positioning of T_RM_ cells in diverse tissues would suggest that tissue-specific adaptations might be required for their long-term maintenance and specific function. Yet, the transcriptional make up of CD8 T_RM_ cells in different tissues is largely similar (90, 91). Lipids are recognized to be an important substrate and FAO as important source of energy for CD8 T_RM_ cells, but the signals resulting the bioavailability of FFA stored in lipid droplets remain to be discovered. Upon activation, CD8 T_RM_ cells appear to use similar metabolic pathways compared with effector CD8 T cells, using OXPHOS and glycolysis. However, maintenance of T cells sets CD8 T_RM_ cell apart, with the high levels of P2RX7 increasing susceptibility to cell death, the expression of AhR critical for survival and the altered cardiolipin composition and mitochondria activity of those CD8 T cells residing at the top layers of the skin and intestine.

The biochemical analysis of T_RM_ cells has been hampered due to the difficulties in harvesting sufficient cell numbers and the inability to culture these cells, requiring constant interactions with tissue cells. Their important role in providing immediate protection against microbial invasion as well as tissue homeostasis and their role in systemic metabolism and pathological conditions, combined with technological advances enabling more sensitive cellular and biochemical analysis, will contribute important new discoveries in the coming years.

## Author Contributions

All authors listed have made a substantial, direct and intellectual contribution to the work, and approved it for publication.

### Conflict of Interest Statement

The authors declare that the research was conducted in the absence of any commercial or financial relationships that could be construed as a potential conflict of interest.
